# Rethinking fish-friendliness of pumps by shifting focus to both safe and timely fish passage for effective conservation

**DOI:** 10.1038/s41598-024-67870-5

**Published:** 2024-08-02

**Authors:** Oliver J. Evans, Josh Norman, Liam J. Carter, Thomas Hutchinson, Andrew Don, Rosalind M. Wright, Jeffrey A. Tuhtan, Gert Toming, Jonathan D. Bolland

**Affiliations:** 1 Hull International Fisheries Institute, University of Hull, Hull, UK; 2 Energy and Environment Institute, University of Hull, Hull, HU6 7RX UK; 3 Environment Agency, Rivers House, East Quay, Bridgwater, Somerset, TA6 4YS UK; 4 Environment Agency, Inworth Road, Rivers House, Threshelfords Business Park, Feering, CO5 9SE UK; 5 Department of Computer Systems, Tallinn University of Technology, Ehitajate Tee 5, 19086 Tallinn, Estonia

**Keywords:** Behavioural response, Catadromous, Entrainment, Fish screening, Multi-beam sonar (ARIS), Operational changes, Ecology, Ecology, Environmental sciences, Hydrology

## Abstract

Globally, catadromous freshwater eels of the genus Anguilla are of conservation concern, including critically endangered European eel (*Anguilla anguilla*). Pumping stations that move river water to a higher elevation severely impact eels during their seaward spawning migration. Fish-friendly pumps can mitigate fish injury and mortality but here we uniquely rethink a fish-friendly pump as a fish passage solution. In this pluriannual study, the seasonal timing of pump operation was misaligned with the typical silver eel migration period. Eels were almost exclusively nocturnal but night-time pumping represented as little as 5.6% a year. Night-time eel approaches were primarily influenced by pump duration and temperature, but did not align with lunar phase, unlike in unregulated rivers. After reaching the pumping station, eel passage was influenced by weedscreen aperture and increased when the aperture was increased. Passive sensor collision suggested non-pump infrastructure could cause injury and mortality to eels. It is therefore recommended pump operation should align with the timing of silver eel migration, weedscreen and pump entrance efficiencies should be maximised, and non-pump infrastructure must have low fish injury risk. Ultimately, considering the entire structure a fish passage solution will help ensure fish-friendly pumps have high conservation value for anguillid eels globally.

Freshwater eels of the genus *Anguilla* are globally distributed in over 150 different countries^1,2^. They have a complex catadromous lifecycle, migrating between freshwater growth habitats and offshore spawning areas, although they are known to inhabit estuarine and coastal habitats^1^. Despite their vast ranging distribution and highly adaptive capacity (e.g. phenotypic plasticity), temperate eel species are experiencing population declines and are of conservation concern^3,4^. The European eel (*Anguilla anguilla*), for example, is classified as critically endangered by the IUCN^5^ following a multi-decadal decline^6^ attributed to a multitude of anthropogenic pressures, including overfishing, pollution, climate change, disease (parasites), habitat loss and barriers to migration^7,8^. Hence, the European Commission (EC) has established legislation (Regulation No. 1100/2007) to reduce anthropogenic mortality and increase the probability of silver (adult) eel escapement to the sea^9^. Consequently, there is an urgent need to identify and study measures to help conserve European eels and anguillid eels more generally.

Pumping stations move river water to a higher downstream elevation for river level management, including flood prevention in Europe, North America, Asia, and Australasia^10–12^. The future requirement for pumping stations will also be exacerbated by climate change^13^ and population growth in low-lying areas^14^. Given the necessity of pumping stations now and in the future, they warrant special attention by conservation practitioners because they have disproportionally severe impacts on anguillid eels. Pumping stations can present a barrier to upstream migrating juvenile eels^15^ and eels that inhabit freshwater habitats upstream will encounter them during obligatory seaward spawning migration^1,16,17^. Yet, pumping stations are complete barriers to longitudinal connectivity when not operational and traditional-style pumps injure and kill fish during operation^10,18–20^.

Fish-friendly pumps which aim to provide > 99% fish survival and no change to natural fish behaviour are becoming increasingly recognised as the preferred management approach to help conserve anguillid eels^21^. Indeed, fish-friendly axial flow pumps have been shown to reduce direct injury and mortality of European eels during passage^22^. Archimedean screws have been used to displace water upwards for millennia, and Archimedean Screw Pumps (ASPs) have recently been developed as a fish-friendly alternative to traditional pumps. ASPs can have a gap around rotating helix and can injure fish by striking and grinding^10,23^. Shrouded ASPs, such as the pump studied here, fully enclose the helix in a rotating ‘support tube’ to eliminate abrasive injuries but their fish-friendliness remains unstudied. Notwithstanding, understanding the overall effectiveness of fish-friendly pumps as a method to ensure anguillid eel conservation must also consider environmental and biological processes before and after fish passage.

A fish passage solution is any device, structure, or mechanism which is designed or operated to facilitate the safe movement of fish in either an upstream or downstream direction past one or several impediments^24^. When present, pumping stations are typically the only downstream passage route, and thus we uniquely propose the entire pumping station should be considered as a fish passage solution. In doing so, the focus will shift from merely the fish-friendliness of the pump to considering if management practices and wider infrastructure influence whether passage occurs. More specifically, seaward migrating adult silver eels must approach the pumping station while operational, which is stochastic depending on rainfall and upstream water levels^12^, and thus may confound other biotic and abiotic (e.g., river flow, temperature, and lunar cycle) influences on the timing of silver eel migration. Therefore, ecologically unfavourable management could degrade the conservation value of fish-friendly pumps as a fish passage solution for anguillid eels.

Seaward migrating eels must then pass through the entire pumping station, including the weedscreen, pump and tailrace infrastructure (e.g., penstock and flap gate), unharmed and without delay to be considered an effective fish passage solution. Although the response of migratory fish to anthropogenic infrastructure remains poorly understood, migratory (silver) European eels are known to retreat from weedscreens^20,25^ and pumps^25^ at traditional (i.e., non-fish-friendly) pumping stations. Elsewhere, non-pump infrastructure, such as spillways at dam outfalls^26^ and over/under shot weirs^27^, have also been demonstrated to injure and kill fish. Thus, further emphasising the need to understand the effectiveness of the entire pumping station as a fish passage solution.

The overall aim of this study was to assess the effectiveness of a pumping station with a fish-friendly shrouded ASP as a fish passage solution for seaward migrating (silver) European eel. A novel combination of sonar and sensor technology were employed to enable the replacement of live fish^28^, which are typically used to assess injury and mortality at both traditional style and fish-friendly pumps^10,18–20^ and hydropower turbines^23,29–31^. The specific objectives were to assess:

- When the pumping station operated, and thus provided an opportunity for downstream silver eel passage,
- When silver eels approached the pumping station on a seasonal and diel basis using multi-beam sonar (ARIS), including the influence of daily total pump duration, daily average river temperature and lunar phase,
- Silver eel behaviour at the weedscreen, including the influence of abiotic (i.e., turbidity) and biotic (i.e., eel speed) variables on rate of passage, (tactile, non-tactile) and non-tactile retreat distance across three different weedscreen apertures,
- Silver eels which passed the weedscreen but retreated from the ASP, and the internal conditions of the pumping station, including a large flap gate to prevent tidal ingress, using passive sensors and previously defined fish injury thresholds for pressure and acceleration.

The novel findings presented in this study will provide insights into the challenges associated with providing safe and timely European eel passage in fragmented rivers with water management structures and will be used to inform management of fish-friendly pumps to help deliver European eel conservation.

## Results

### Pumping station operation (i.e., European eel downstream passage opportunity)

**Figure 1:**
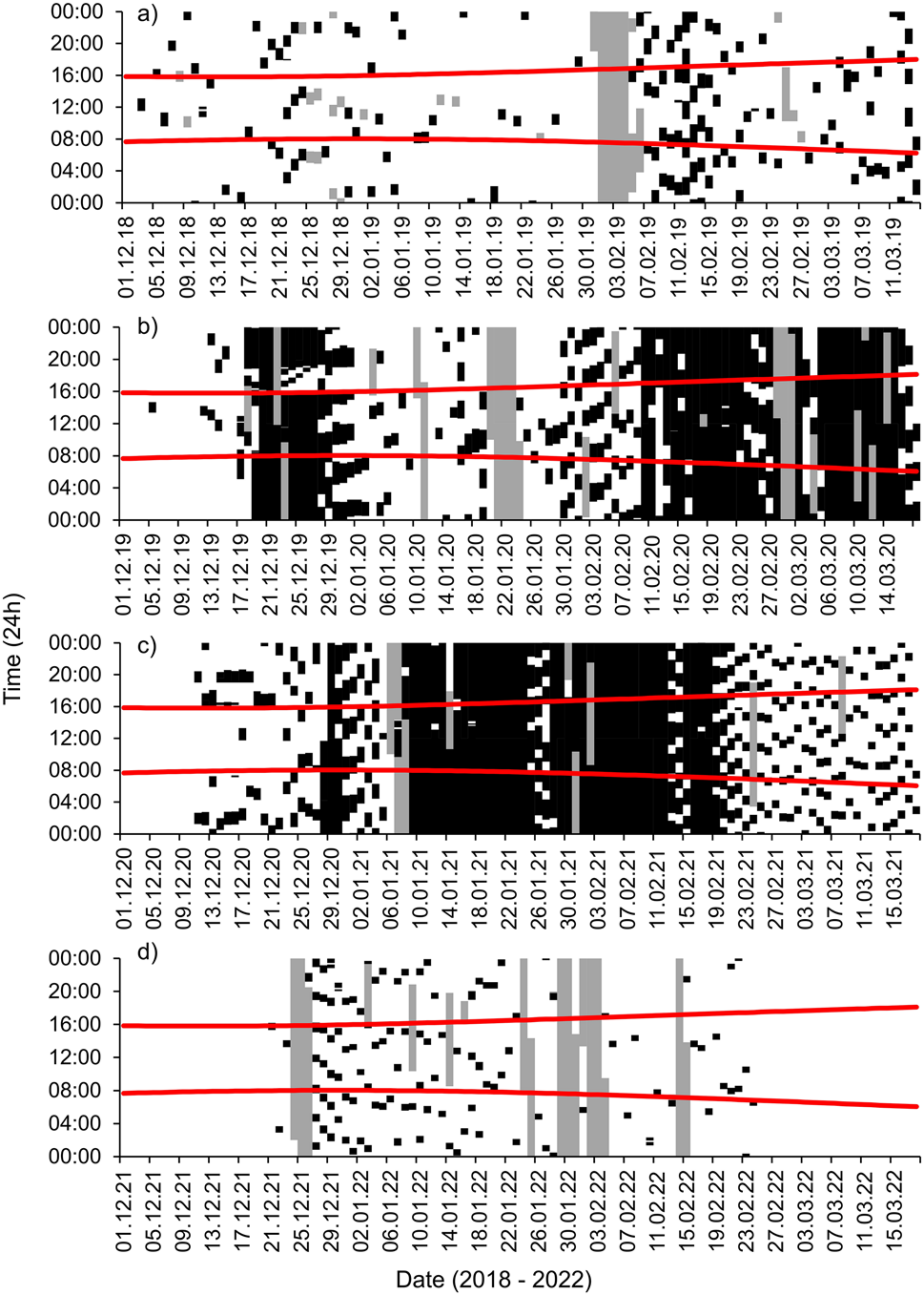
Daily pump operation (black pixels) between 1 December and 18 March in 2018/19, 2019/20, 2020/21 and 2021/22 (top to bottom), including sunrise/sunset (red lines) and missing data (grey pixels).

The pumping station first operated in December in all four study years. The amount the pumping station operated was highly variable between years; very little in Y1 and Y4 (night-time pump operation = 5.6% of the total night hours per year) in comparison to Y2 and Y3 (night-time pump operation = 56.5% of the total night hours per year), which had far more (Table 1). The timing of pump operation per day was also intra- and inter- annually variable (Fig. 1).

**Table 1:** Summary of pumping station operation during each study year (01/12 – 18/03).

Metric	2018/19	2019/20	2020/21	2021/22
Pump first operated (*n* days studied)	03.12.2018 (102)	05.12.2019 (105)	11.12.2020 (98)	21.12.2021 (66)
First pumped > 4 h per day	22.12.2018	17.12.2019	12.12.2020	27.12.2021
Total hours pumping (h:mm)	204:38	1125:53	1287:59	90:19
Total both pumps operating (h:mm)	00:00	140:54	286:34	00:00
Mean ± SD pump hours per day (h:mm)	2:00 ± 02:21	10:43 ± 08:57	13:09 ± 08:45	01:23 ± 01:23
Hours of night pumping (% of night hours during study period) (h:mm)	119:17 (7.9%)	653:57 (42.2%)	811:50 (56.5%)	56:06 (5.6%)
Julian days with no pump operation (*n*) (% study period)	28 (27.5%)	15 (14.3%)	1 (1.0%)	13 (19.7%)
Nights with no pump operation (*n*) (% study period)	47 (46.1%)	26 (24.8%)	2 (2.0%)	23 (34.9%)
Julian days when pump operated for 24 h (%)	0	13	28	0

### European eel approach to the pumping station

**Figure 2:**
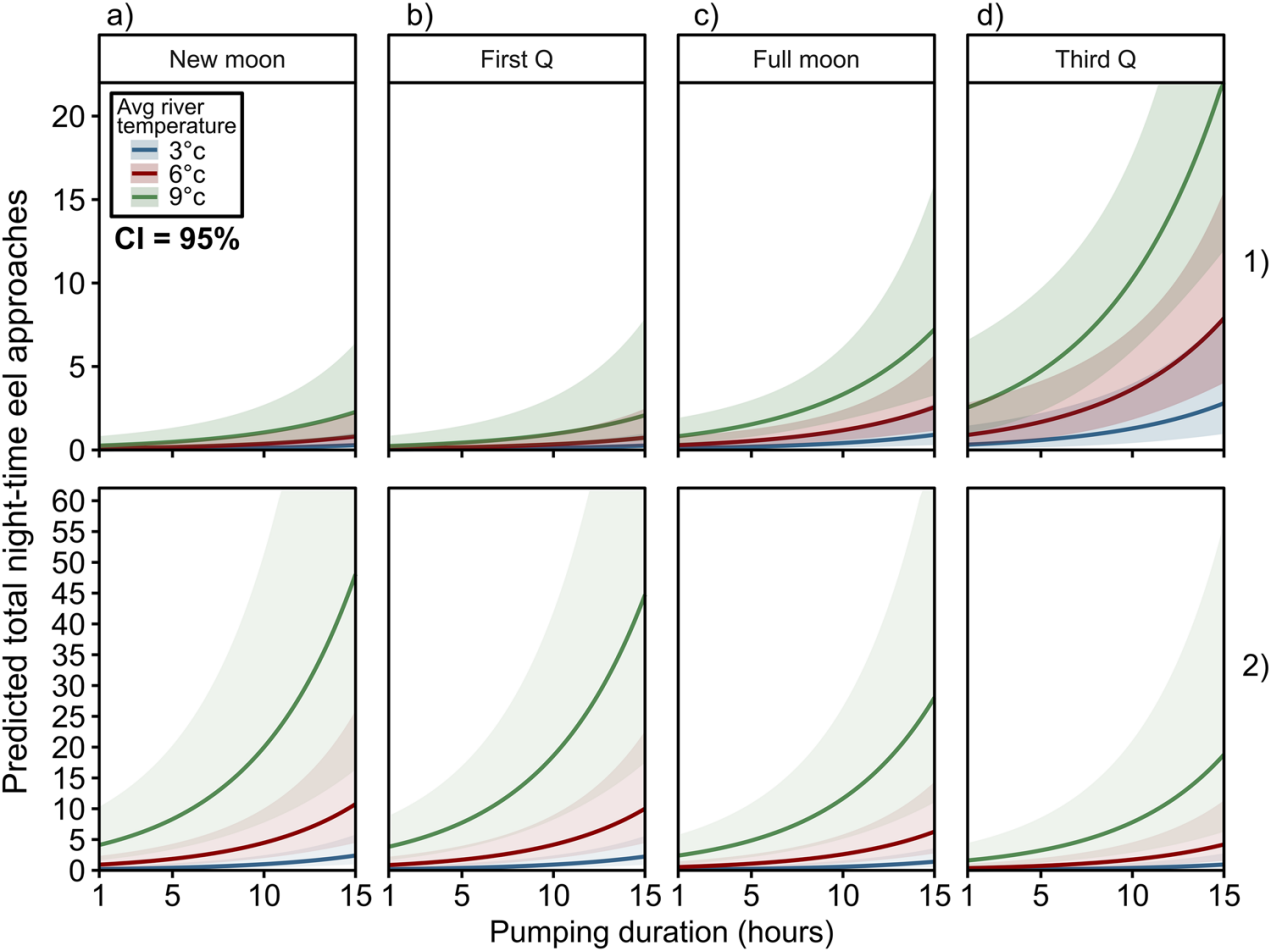
The predicted effect of pump duration on total number of night-time eel approaches at Bells pumping station in 2019/20 (1) and 2020/21 (2) (facetted by lunar phase **a**–**d**). Negative binomial (zero inflated) lines for each temperature class fitted by the GLMM (Table S4). 95% confidence intervals represented by shaded envelope surrounding smoothed line (upper and lower bounds).

A total of 256, 157, 424 and 185 eels approached the pumping station in Y1–Y4, respectively (Table S1). Eels were almost exclusively nocturnal, with 96.1–98.9% of eels approaching between sunset and sunrise each year, despite 37.9–41.9% of the pump operation being during the day (Figs. S1 and S2). The results from the GLMM suggested night-time eel approaches to the pumping station were primarily influenced by pump operation duration but this was also significantly influenced by lunar phase; eel approaches were predicted to be highest during the third quarter and new moon phases in Y2 and Y3, respectively (Fig. 2.1d, 2.2a; Table S4). Temperature overall had an additive effect where night-time eel approaches were predicted to increase linearly with increasing temperature (Fig. 2; Table S4). Thus, night-time eel approaches were highest when it was warmer, and when pumps operated for longer.

### European eel behaviour at the weedscreen

After eels approached the pumping station the primary driver of passage probability was weedscreen aperture; passage probability significantly increased from 36% (100-mm aperture) to 52% (190-mm aperture) (Fig. 3.1a; Table S5). Passage probability was then influenced significantly by a positive linear relationship with eel speed in which the predicted passage probabilities increased from 29 to 45% (100-mm aperture), 46–63% (212-mm aperture) and 44–60% (190-mm aperture) as eel speed increased from 0.2 to 0.5 ms^−1^ (Fig. 3.1b; Table S5). Turbidity did not significantly influence passage probability but an interaction between eel speed and turbidity was close to significance (*p* = 0.09); passage probability marginally increased with eel speed at high turbidity (50 NTU) but not at low turbidity (10 NTU) (Fig. 3.1c; Table S5).

**Figure 3:**
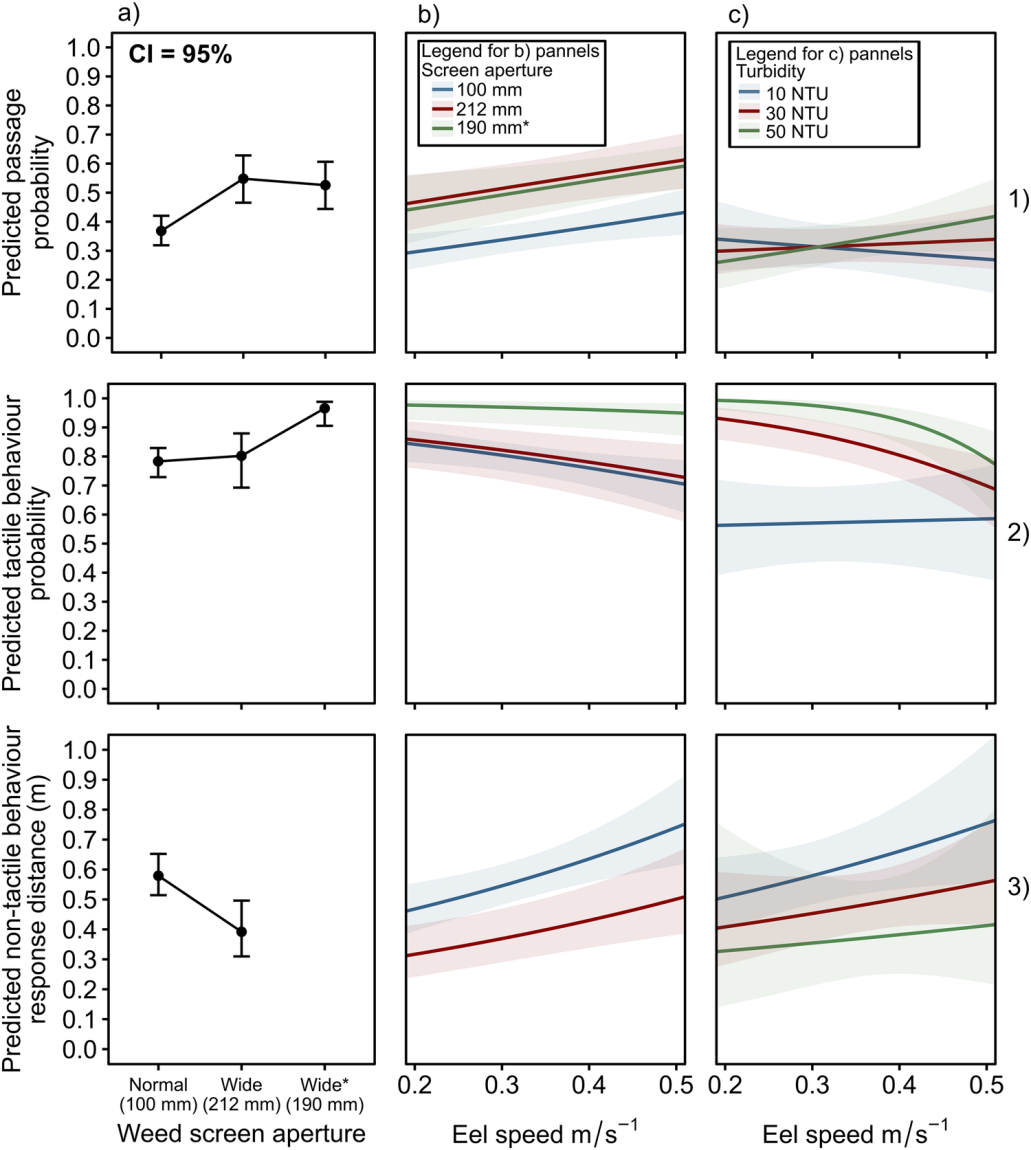
Derived from European eels which approached the pumping station; the predicted probabilities for (1) passage at the weedscreen, (2) tactile behaviour of eels which don’t pass and (3) non-tactile response distance for eels which have non-tactile response in 2019/20 (Y2), 2020/21 (Y3) and 2021/22 (Y4). (**a**) panels predict the influence of screen aperture, with * denoting when the entire screen was replaced with 190-mm aperture (insufficient data for non-tactile response distance at w190-mm aperture. (**b**) panels show represent eel speed across screen apertures, and (**c**) panels show the interaction between eel speed and turbidity. Negative binomial (zero inflated) lines (panels 1, 2) fitted by the GLMMs chosen in the model selection process (Tables S1, S2, S5, S6). Gamma lines (panel 3) fitted by GLM (Tables S3, S7). 95% confidence intervals represented by shaded envelope surrounding smoothed line (upper and lower bounds).

When eels approached but did not pass the weedscreen, the probability of tactile retreat behaviour (e.g., touching the weedscreen) was again influenced by weedscreen aperture and eel speed (Fig. 3.2; Table S6). Tactile behaviour probability significantly increased from 78% (100-mm aperture) to 96% (190-mm aperture) (Fig. 3.2a; Table S6). Tactile behaviour probability was then influenced significantly by a small negative linear relationship with eel speed in which the predicted tactile behaviour probabilities decreased from 84 to 68% (100-mm aperture) and 85–70% (212-mm aperture) as eel speed increased from 0.2 to 0.5 ms^−1^ but had no influence at 190-mm aperture (Fig. 3.2b; Table S6). Turbidity was significantly positively correlated with tactile behaviour probability but only for slow speed eels (0.2 ms^-1^) (Fig. 3.2c; Table S6).

When eels retreated before touching the weedscreen (e.g., non-tactile), the predicted non-tactile response distance was significantly higher at 100-mm aperture (0.57 ± 0.05 m) compared to 212-mm screen aperture (0.39 ± 0.11 m) and was positively correlated with eel speed (Fig. 3.3a,b; Table 7). Increasing turbidity appeared to be negatively correlated with non-tactile response distance but this relationship was not significant nor was a turbidity and eel speed interaction (Fig. 3.3c; Table S7).

### Retreat from the Archimedean Screw Pump

A total of 33, 48, 100 and 29 eels in Y1 – Y4, respectively, were observed swimming back through the weedscreen in an upstream direction, i.e., retreated from the ASP, which equated to 12.9%, 30.6%, 23.6% and 15.7% of those that approached and 31.7%, 70.6%, 60.2% and 27.1% of those that passed through the weedscreen, respectively.

### Internal conditions of the pumping station

**Figure 4:**
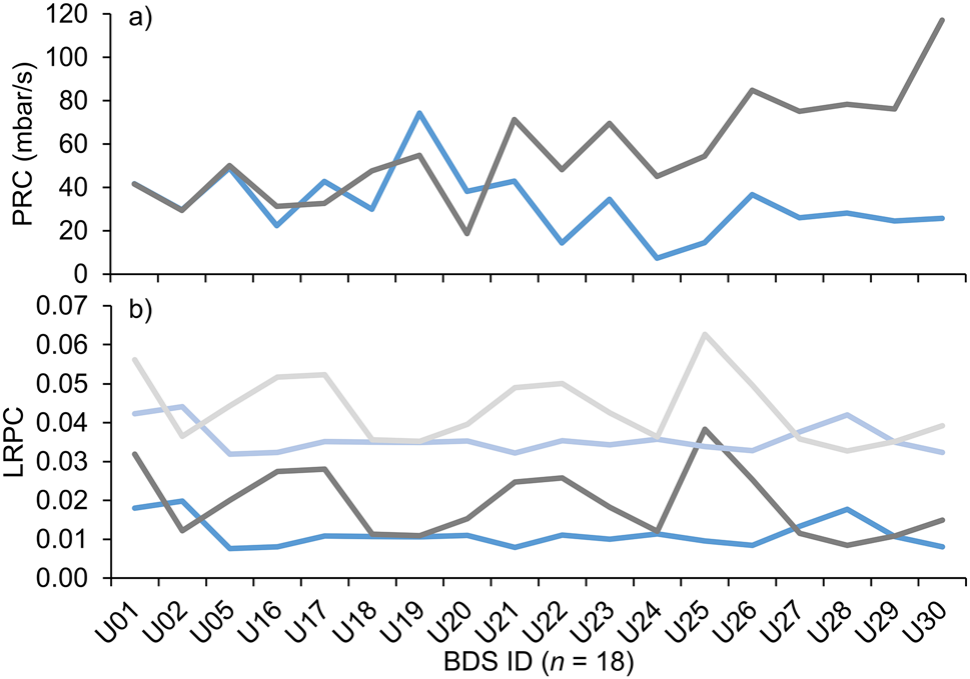
Pressure rate of change (PRC: (**a**) and minimum and maximum log ratio of pressure change (LRPC: (**b**) comparison between in screw (blue) and screw exit to tailwater (grey) for BDS (n = 18) where the top lines (light shading) represent maximum LPRC (e.g., higher acclimation depth) and bottom lines (darker shading) represent minimum LPRC (e.g., lower acclimation depth).

Nadir (mean ± SD) (mbar) during BDS passage was 974.50 ± 18.60 (range 933.40–1000). The PRC (mean ± S.D) (mbar/s) within the screw was 32.38 ± 14.80 (7.39–74.33), which was significantly lower than 57.01 ± 23.56 (18.64–117.10) for screw exit to tailwater (Paired t-test, t = -3.6444, df = 34, *p* =  < 0.001) (Fig. 4). Likewise, the median (Interquartile range, range) minimum and maximum LRPC for the screw were 0.011 (0.003, 0.008–0.020) and 0.035 (0.003, 0.032—0.041), which were significantly lower than 0.017 (0.014, 0.008—0.038) (Wilcox test, Z = 14, *p* =  < 0.001) and 0.041 (0.014, 0.033–0.063) (Wilcox test, Z = 14, *p* =  < 0.001) for screw exit to tailwater, respectively (Fig. 4).

**Figure 5:**
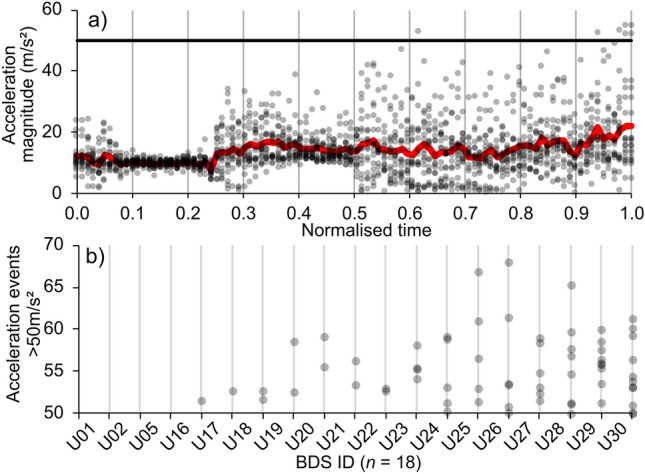
Acceleration magnitude between injection and tailwater for all sensors during normalised passage with acceleration injury threshold (black line) and mean acceleration of all sensors (red line) (**a**) and count of acceleration events (> 50 m/s^2^) recorded by each BDS (n = 18) between ASP exit and the tailwater ordered from least to most events (**b**).

The largest acceleration recorded was 68.0 m/s^2^ and all acceleration events > 50 m/s^2^ (*n* = 66) occurred in the ‘screw exit to tailwater’ region, i.e., there were none when entering or passing through the ASP. The number of events per BDS was 3.8 ± 3.3 (range 0–11) (Fig. 5).

## Discussion

Here, we have demonstrated that modern fish-friendly pumps do not necessarily equate to a rotating fish passage solution to conserve a critically endangered fish species, the European eel. Given their catadromous lifecycle, eels must migrate from freshwater habitats to ultimately spawn at sea, but pumping stations are complete barriers to longitudinal connectivity when not operational. When operational, eels must pass without substantial delay or injury, but pumping station operation here was highly stochastic depending on rainfall and upstream water level. Thus, although large numbers of European eels approached and passed through the pumping station during the wettest years, it represented an ineffective fish passage solution during dry periods with infrequent pump operation and daytime operation. Notwithstanding, eels were observed retreating from the weedscreen and pump even when it was operating during ecologically favourable periods. Therefore, our findings propose that a combination of traditional management procedures and the effect of non-pump infrastructure could degrade the value of fish-friendly pumps as a tool to deliver anguillid eel conservation.

When a fish-friendly pumping station (with no alternative passage route) is not operational, no downstream passage route is available, and the system is effectively closed. Across Europe, silver eels typically emigrate from non-pumped rivers in late summer to early winter, although variable between years. For example, over 40 years of data from Irish and Norwegian rivers were used to establish that migration onset varied between July/August and October and ceased between October and December^32^. Here, the pumping station was not operational until December and thus up to five months of eel migration could have been missed due to lack of passage opportunity. There was also large inter- and intra-annual variation in the amount of pump operation which significantly influenced the number of eels that approached the pumping station; as few as 90.3 h of pump operation in 2021/22 when 185 eels were imaged and as many as 1288 h in 2020/21 when 424 eels were imaged. Eels approached the pumping station as late as mid-March, which is later than most rivers in Europe, but ‘spring runs’ have been reported^33,34^. In the future, climate change will lead to more extreme droughts and greater pressures/demands (i.e., abstraction/withholding of water) on water resources, and thus has potential to shift pump operation and push the onset of eel migration in pumped catchments to even later in the winter.

Daily total pump duration (associated with increased rainfall and likely elevated turbidity) and temperature significantly increased pumping station approach. Elsewhere, eels are known to migrate during periods of elevated flow^10,35^ and the influence of temperature on eel movement has also been reported^36,37^. However, pumping station approach was independent of the lunar phase, which contrasts with unregulated rivers where eels migrate during the new moon phase of the lunar cycle in the absence of elevated flow^32,38,39^, and thus such escapement opportunities were invariably missed here. Furthermore, eels were almost exclusively nocturnal^20,35,40^, and thus daytime pump operation can be regarded as wasted passage opportunity, especially during dry periods with infrequent pump operation. For example, pumps operated for as few as 90 h in 2021/22 (5.6% of the study period) and 37.9% of pump operations were during the day but accounted for only 1.1% of eel approaches. Ultimately, pump operations were misaligned with European eel migration and thus prevented the fish-friendly pump from being an effective fish passage solution. It is hence recommended that operations are modified, as has been performed at pumping stations with gravity sluices to provide safe downstream passage for silver European eels^41^ and protect river-resident fish^42^.

Eels were reluctant to pass through the original weedscreen but increasing the weed screen aperture influenced eel behaviour and improved overall night-time passage rate, and thus was a fundamental advancement in making the entire pumping station an effective fish pass solution. Biotic (swimming speed) and abiotic (turbidity) variables also had a significant influence on the behaviour, but less so than screen aperture. Tactile response probability was positively correlated with turbidity whereas non-tactile response distance was negatively correlated, which suggests visual cues were important to avoid screen contact, similar to elsewhere^43^. However fish are known to use different sensory systems to detect and avoid fish screens in the presence or absence of light^44^. Notwithstanding, over 40% of eels retreated from the widest bar spacing; weedscreen aperture, orientation, bar shape and material can affect eel behaviour^45–47^, and thus could be modified to further increase the passage rate. In addition, up to 70% of eels that had passed through the screen subsequently passed back upstream, presumably after approaching the ASP, as reported elsewhere^25^. Retreat may have been caused by the rotating scoop-like action of the ASP creating complex flow fields or possibly noise created by pumps^48^. Indeed, understanding the influence of infrastructure on movement behaviour of fish into and through fish passage solutions is a global challenge for conservation of migratory fish^49^.

Non-operation until December, infrequent passage opportunities and retreat from the weedscreen and ASP all induced migration delays. Such delays can have huge negative impacts on migrant eels as they are more prone to predation^20,50^ and deplete energy reserves required to complete their seaward migration^37,51^. Substantial delay may cause these migrants to miss the seasonal spawning in the Sargasso Sea^17^, and thus not contribute to the breeding population nor future generations.

When designing engineered conservation solutions, such as fish-friendly pumps, greater emphasis needs to be placed on the entire structure being fish-friendly. Here, passive sensors revealed the risk of blade strike and pressure related injuries were extremely low, based on laboratory assessments with American eels^52,53^. However, acceleration events (up to 68.0 m/s^2^) over the > 50 m/s^2^ threshold^54^ occurred when the sensors exited the screw and at the flap gate, i.e., non-pump infrastructure may injure eels but higher injury thresholds have been proposed^53,55^. Indeed, further laboratory and field-based research to establish the relationship between sensor values and fish injury/mortality is urgently needed. Notwithstanding, the findings emphasise the value of incorporating state-of-the-art sensor technology to glean a greater understanding of conditions during passage through pump and non-pump infrastructure.

## Conclusions

This multi-year study at a modern fish-friendly pump with traditional operating regime demonstrated a more holistic and ecologically focused approach is required to effectively conserve critically endangered European eel. There was a low risk of injury but there were wider operational and infrastructure considerations to make the entire pumping station a highly effective (rotating) fish pass. Further research is required to validate these findings at similar pump infrastructure, globally, but the following is recommended for fish-friendly pumping stations to provide both safe and timely passage:

1. Fish-friendly pumping stations must align pump operation with when silver eel migrate in unregulated rivers; at night during new moon phase of the lunar cycle between September and February for European eel. During periods of water scarcity (e.g., early autumn), this recommendation may create conflicts with other water stakeholders elsewhere in the catchment, such as for agricultural irrigation, and thus additional approaches to conserve water and minimise abstraction rates may be required. Failing that, alternative downstream passage routes may need to be provided for silver eels despite the installation of fish-friendly pumps. Conversely, pumps should only operate during the day for flood prevention to avoid wasting night-time passage opportunities. River levels should be temporarily elevated (without increasing flood risk) prior to a new moon to also simulate a flood and maximise pumping duration^41^.
2. Weedscreen and fish-friendly pump passage rates must be maximised. Notwithstanding, the primary functions of a weedscreen, i.e., debris management to protect pumps and prevent people access to the sump, cannot be compromised.
3. Non-pump infrastructure (e.g., flap gate) that could injure fish should be replaced with fish-friendly equivalents but requires injury threshold validation^53^.

Overall, the findings from this novel research demonstrate a step change in the way we think about fish-friendliness and how we operate anthropogenic infrastructure (i.e., pumping stations and hydropower facilities) is urgently required, rather than merely considering the likelihood and consequence of pump injury. Traditional management practices were misaligned with the seasonal, lunar, and circadian, migratory rhythms and behaviour of critically endangered European eel. In the future, ecological knowledge of the target species should be incorporated into operating regimes and physical design of anthropogenic infrastructure to maximise escapement. Doing so will require strategic planning, including considering trade-offs between water management practices and fish conservation, and multi-objective assessment of performance predicated on progressive conservation targets. Failing to do so will negate the potential conservation gains from highly expensive engineered solutions. Ultimately, by considering the entire structure a fish passage solution the definition of effectiveness is far more comprehensive, and thus will help ensure fish-friendly pumps are of high conservation value for anguillid eels globally.

## Materials and methods

### Study site

**Figure 6:**
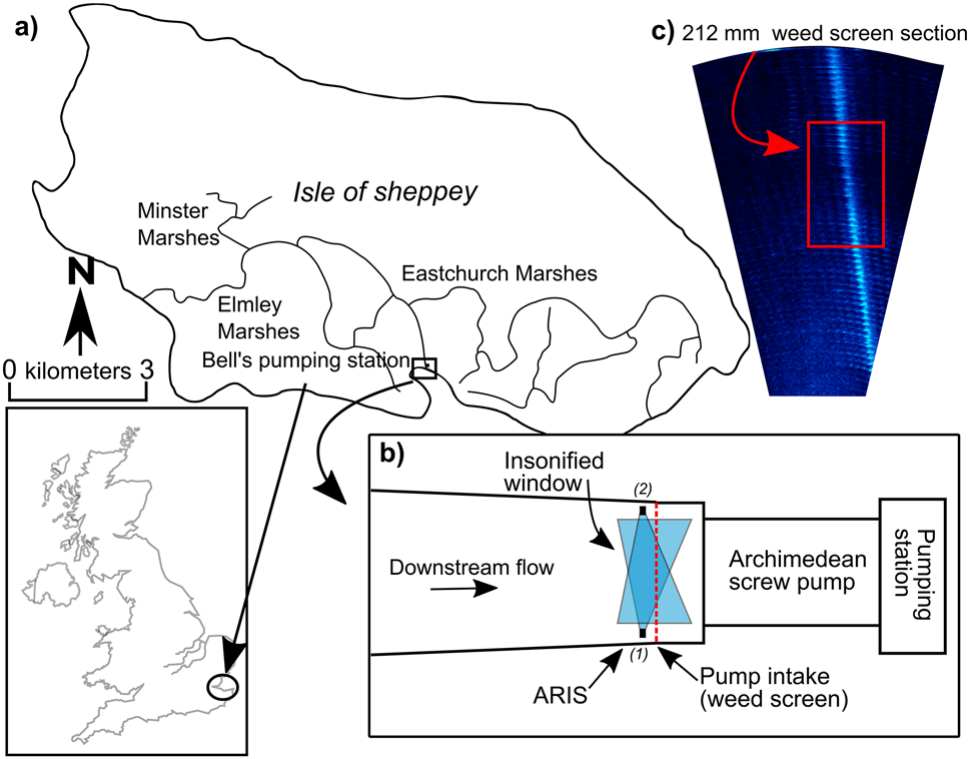
The location of Isle of Sheppey within the UK (**a**), a schematic of Bells pumping station, and the multi-beam (ARIS) insonified window across the channel in years one–three (1) and year four (2) (**b**), and screenshot of ARIS output with 1-m section of 212-mm wider screen aperture (red rectangle) (c).

Bells pumping station (51° 2″ 13.5″ 0°5″ 24.2″ E) is located on the Isle of Sheppey and provides flood protection to 34km^2^ catchment (Fig. 6a). No eel population data, via either entire fish community or eel-specific monitoring surveys, was available for the upstream catchment. No eel stocking had (knowingly) been performed, but the upstream passage route into the catchment was unquantified. The pumping station has two shrouded ASPs, each with a 2.5 m diameter and 10.5 m long helix with 3 blades, run at 11.2–23.3RPM, and have a 1750L/s capacity. The pumping station was fronted by a 10 m long weedscreen (bar thickness 12-mm) with 100-mm apertures in 2018/19 (Y1). In 2019/20 (Y2) and 2020/21 (Y3), four bars were removed from the weedscreen to create a 1-m section with 212-mm apertures, and in 2021/22 (Y4), the entire weedscreen was replaced with 190-mm apertures (10-mm bars). A water quality monitor (YSI 6600 Sonde, Xylem Analytics, USA) was positioned ~ 25 m upstream of the study site and was used to collect the abiotic variables temperature (**°**C) and turbidity (Nephelometric Turbidity Units: NTU) in all study years except Y4.

### Multi-beam sonar

Acoustic cameras, such as Adaptive Resolution Imaging Sonar (ARIS; Soundmetrics, Belleview, USA), enable passive fish monitoring and are more efficient than net-based methods during low light and highly turbid water^56^. An ARIS Explorer 1800 was used in this study to collect data on eel approach, passage, and retreat behaviour at Bells pumping station. It is not possible to distinguish between European eel life stages (i.e., yellow and silver) with ARIS images and yellow eels can also move between habitats, but imaged eels were assumed to be silver given the timing of the movements. The ARIS was installed on 12.11.2018, 08.10.2019, 04.11.2020 and 09.12.2021 collecting 128-, 162-, 146- and 77-days footage in respective years. The ARIS operated in high frequency mode (1.8 MHz; 28° × 14° beams, 512 bins) with a window length of 10 m (starting 0.7 m from point of transducer) at 8–12 frames s^−1^ (fps), receiver gain at default and focus set to auto. Continuous observations were captured to a 4 TB external HDD which was exchanged throughout the study period. Files were time and date stamped (hh:mm:ss–d/m/y) and stored in 10 min intervals. The ARIS insonified five metres of the 10-m wide weedscreen upstream of pump one (Y1–Y3; Fig. 6b) and pump two (Y4; Fig. 6b), ensuring modified screen apertures were within view (Fig. 6c).

### Passive sensors

Passive sensors (Barotrauma Detection System; BDS) were used to record conditions fish may experience during passage through Bells pumping station^57^. BDS are an underwater passive sensor which measures the total water pressure (mbar), linear acceleration (m/s^2^) and rotation rate (rad/s), which enable analysis of decompression, collisions, and large-scale turbulence^58^.

**Figure 7:**
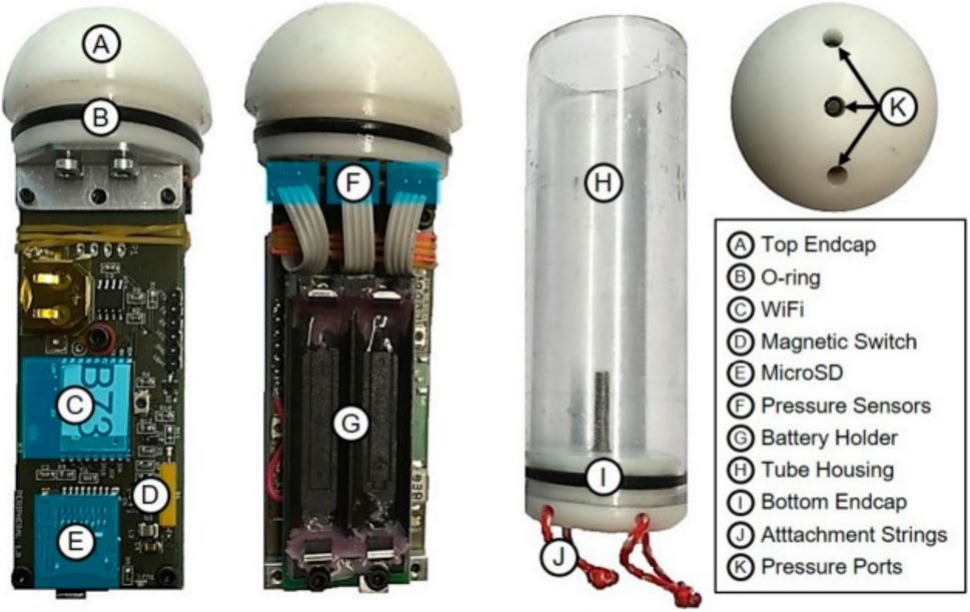
Overview of the BDS. The top endcap (**A**, **B**) contains three pressure transducers (**F**, **K**). Below there are two electronics boards containing the Wi-Fi module I, magnetic switch (**D**), microSD (**E**), and AAA battery holder (**G**). The sensor and electronics payload (**A**–**G**) is inserted into the plastic tube housing (**H**) and screwed by hand onto the bottom endcap (**I**), which also includes two rugged nylon attachment strings (**J**). [Reproduced from^23^].

The custom waterproof BDS housing (polycarbonate tube with end caps: length 140 mm, diameter 40 mm, dry weight 147 g) contains the BDS electronics and two AAA batteries. Inside the hemispherical end cap (Fig. 7) are three identical digital total pressure transducers (Fig. 7). The total pressure consists of three main components: the atmospheric, hydrostatic, and hydrodynamic pressures. The pressure transducers (MS5837-2BA, TE Connectivity, Switzerland) have a maximum pressure of 2000 mbar and provide sensitivity of 0.02 mbar (~ 0.2 mm water column). Data are stored on a microSD card at 0.01 mbar measurement resolution with 1 mbar (~ 10 mm water column) accuracy. A magnetic switch activates the BDS, and the local atmospheric pressure is auto calibrated with all three transducers set to a reference value of 1000 mbar at local atmosphere. This autocalibration allows for BDS data to be compared without effects of local atmospheric pressure at all field and laboratory sites. The BDS also contains a digital nine degree of freedom (DOF) inertial measurement unit (IMU; BNO055, Bosch Sensor Tec, Reutlingen, Germany). The IMU includes a linear accelerometer (range set to ± 8 g / ± 78.45 m/s^2^), gyroscope (range set to ± 2000°/s), and magnetometer (x-, y-axis =  ± 1300; z-axis =  ± 2500 (± 5)). All variables are saved at 100 Hz with exception of the magnetometer, which records data at 20 Hz. Data are downloaded from the BDS via WIFI.

BDS (no changes to original buoyancy) were deployed by hand through the weedscreen upstream of pump one and were recovered using a net (7000_×_2300_x_2300mm length_x_width_x_height) attached to the outfall using ratchet straps. 20 individual BDS were deployed once over two days in consecutive weeks; five on 02.03.2022 (recovered = four, contained passage data = three) and 15 on 10.03.2022 (all recovered and contained passage data). The pump was operated for ~ five minutes before and ~ 15 min after BDS deployment.

### Data analysis

#### Multi-beam sonar analysis: European eel approach, passage, and retreat behaviour

To determine the number of eels which approached the pumping station, and their passage and retreat behaviour at the weedscreen (i.e., Fig. 6c), sonar footage was manually reviewed minute-for-minute by an experienced reviewer using the ARIScope software (v2-8-0). The sonar data was reviewed only when pump one, or both pumps one and two were operational (insufficient spatial coverage for only pump two). European eels were identified from their elongated body shape and sinusoidal swimming^56^ and their size (m; measured using fish measurement tool in ARIScope), approach, passage, and retreat behaviour metrics were recorded (Table S10).

#### Passive sensor analysis: conditions experienced during pump passage

The likelihood of pressure related injuries to occur in live fish was calculated using the passive sensor event data (Table S11) and raw pressure data. The Pressure Rate of Change (PRC) was calculated by subtracting the nadir from the largest pressure reading within the previous one second. The minimum (river bottom; 0.20 m) and maximum (river surface; 0.80 m) Log Ratio Pressure Change (LRPC) was calculated as log(total acclimation pressure/nadir). The total acclimation pressure was the local atmospheric pressure added to water pressure (p) which was estimated using the following equation.

$${\text{water}}\,{\text{pressure}}\,\left( {\text{p}} \right) = \left( {{\text{rho }}*{\text{ g }}*{\text{ h}}} \right)/100$$

rho = density of water (997.05 kg/m^3^,g = gravitational constant (9.81 m/s^2^).h = acclimation depth in m (0.20 and 0.80 m).

During this study, threshold for PRC was ~ 3950 mbar/s^52^ and LRPC was 3.06^53^ based on a lack of mortality and injury during laboratory investigations on American eel (*Anguilla rostrata* (L.)). Increased acceleration during passage through pumping stations have the potential to injure fish; > 50 m/s^2^ has the potential to break fish vertebrae^54^, and thus was the threshold used here.

### Statistical analysis

Using the observed data (Tables S8 and S9), individual Generalised Linear Mixed Models (GLMMs) were built to analyse the European eel approach, passage, and retreat (tactile/non-tactile and response distance) behaviour data in Y2, Y3 and Y4 (R function glmmTMB, package ‘glmmTMB’). Y1 data were insufficient for modelling and only night-time pump operations were modelled because eels were nocturnal.

Total night-time eel approaches (per Julian day) were predicted by including the fixed effects daily total pump duration, daily average river temperature and lunar phase. Sample month was included as a random effect to account for non-independence in eel approaches. We defined the model formula a *priori* and did not use model selection and approach counts were too low in Y4 to model. Variance was homogeneous between sample years, but we modelled Y2 and Y3 independently as annual data were insufficient to include as a random term or interaction with lunar phase and could not represent the influence of contrasting pump operations on night-time eel approaches. Although total night-time eel approach was a count, Poisson models were over dispersed (dispersion > 1.2) and were improved using zero-inflated negative binomial error distributions.

Using data from eels that approached the pumping station, passage probability at the pump (0 = retreat, 1 = pass) was predicted by two binomial (link = logit) GLMM’s including the fixed effect of weedscreen aperture, eel speed (during approach and prior to response, m/s^2^; Table S10) and eel size (m) (Y2–Y4) and turbidity (Y3 only). Because we were interested in eel behaviour once they had approached the pump, we were able to combine annual data (Y2–Y4) using the random effect of year. Eel speed values < 0.19 and > 0.60 were removed (9%) due to heterogeneity across grouping variables year and screen aperture. We used model selection to determine significant predictors of passage probability, with the best model determined by Akaike Information Criterion (AIC) containing screen aperture and eel speed (Y2–Y4). Adding an interaction between screen aperture and eel speed only reduced AIC by 1 and therefore we selected the simpler model (Table S1). For the turbidity model (Y3 only) we included screen aperture and an interaction between eel speed and turbidity (Table S1).

Using data from eels which retreated from the weedscreen, the same fixed and random effects and model selection (as passage probability) were applied to predict tactile behaviour probability (0 = non-tactile, 1 = tactile) (Table S2). Although Δ AIC in the turbidity model (Y3 only) showed no difference in the model containing screen aperture, we maintained the term for parity with the passage models (Table S2). When eels had a non-tactile response, we used the same approach (as tactile behaviour probability) to predict non-tactile response distance (m), except there were few non-tactile responses in Y4 so we removed the random effect of year and only used Y2 and Y3 data. Although the dependant variable was continuous and normal, GLMs fitted with a Gaussian (link = log) distribution had a poor fit and instead we used Gamma (link = log) distributions (Table S3).

For all models we used a standardised approach of applying Levene’s test (R function ‘leveneTest’ in package ‘car’) to check homogeneity of variance, calculating the Variance Inflation Factor (R function ‘vif’ in package ‘car’) to check for multicollinearity and Shapiro–Wilk tests for normality (R function ‘shapiro.test’ in package ‘stats’). Additionally, model validation (R function ‘testDispersion’, ‘testZeroInflation’ and ‘simulateResiduals’ in package ‘DHARma’) was used to select error distributions e.g., zero-inflation, and examine residuals. The passive sensor data had mixed distributions and thus both paired t tests (R function ‘t.test’ in package ‘stats’) and Wilcox rank sum (R function ‘wilcox.test’ in package ‘stats’) were used to investigate internal conditions in the pumping station by comparing differences in PRC and LRPC between within screw and screw exit to tailwater passage. All data were analysed using R version 4.3.1^59^ in RStudio 2023.06.0^60^.

### Supplementary Information

Supplementary Information.

## Data Availability

The data that support the findings of this study are available from the corresponding author upon reasonable request.
