# Supplementary material for: Rethinking fish-friendliness of pumps by shifting focus to both safe and timely fish passage for effective conservation
Source: Sci Rep. 2024 Aug 2;14:17888. doi: 10.1038/s41598-024-67870-5 (PMC11297292; doi:10.1038/s41598-024-67870-5)
Supplement: Supplementary file 1 — Supplementary Information. [file 41598_2024_67870_MOESM1_ESM.pdf]

Title

Rethinking fish-friendliness of pumps by shifting focus to both safe and timely fish passage for effective conservation

Author names and affiliations

Oliver J. Evans[1,2], Josh Norman[1], Liam J. Carter[1], Thomas Hutchinson[1], Andrew Don[3], Rosalind M. Wright[4], Jeffrey A. Tuhtan[5], Gert Toming[5], **Jonathan D. Bolland[1]***

[1] Hull International Fisheries Institute, University of Hull, UK

[2] Energy & Environment Institute, University of Hull, Hull, HU6 7RX[3] Environment Agency, Rivers House, East Quay, Bridgwater, Somerset, TA6 4YS, UK

[4] Environment Agency, Rivers House, Threshelfords Business Park, Inworth Road, Feering CO5 9SE, UK

[5] Department of Computer Systems, Tallinn University of Technology, Ehitajate tee 5, 19086 Tallinn, Estonia

Corresponding author

Email: J.Bolland@hull.ac.uk

Supplementary tables and figures

Table S1 Alternative model selection (Generalised Linear Mixed Models; logit-linked binomial distribution) to determine passage probability of European eels which approached the pumping station. For each model the associated Akaiki Information Criteria (AIC) were calculated using glmmTMB in R 4.3.1. Light grey shading denotes model selected.

Model	Model formula	AIC	Model compared
Screen type + biotic variables Y2 – Y4 (2019/20 – 2021/22)			
mod1_b	passage ~ screen_type*eel_speed+~ screen_type*eel_size + (1|year)	926	(null - mod1)
mod2_b	passage ~ screen_type*eel_speed+(1|year)	923	(mod1- mod2)
mod3_b	passage ~ screen_type+eel_speed+(1|year)	924	(mod2- mod3)
mod4_b	passage ~ screen_type+(1|year)	929	(mod2- mod4)
mod5_b	passage ~ eel_speed +(1|year)	937	(mod2- mod5)
mod6_b	passage ~ eel_size +(1|year)	946	(mod2- mod6)
Null_b	passage ~ 1	952	-
Screen type + abiotic variables Y3 (2020/21)			
mod1_ab	passage ~ screen_type+eel_speed*turbidity	478	(null – mod1)
mod2_ab	passage ~ eel_speed*turbidity	487	(mod1 – mod2)
Null_ab	passage ~ 1	487	-

Table S2. Alternative model selection (Generalised Linear Mixed Models; logit-linked binomial distribution) to determine tactile behaviour probability of European eels which retreated from the pumping station. For each model the associated Akaiki Information Criteria (AIC) were calculated using glmmTMB in R 4.3.1. Light grey shading denotes model selected.

Model	Model formula	AIC	Model compared
Screen type + biotic variables Y2 – Y4 (2019/20 – 2021/22)			
mod1_b	retreat_response~ screen_type*eel_speed+screen_type*eel_size + (1|year)	389	(null - mod1)
mod2_b	retreat_response~ screen_type*eel_speed+(1|year)	390	(mod1- mod2)
mod3_b	retreat_response~ screen_type+eel_speed+(1|year)	386	(mod2- mod3)
mod4_b	retreat_response~ screen_type+(1|year)	391	(mod3- mod4)
mod5_b	retreat_response~ eel_speed +(1|year)	392	(mod3- mod5)
mod6_b	retreat_response~ eel_size +(1|year)	397	(mod2- mod6)
Null_b	retreat_response~ 1	398	-
Screen type + abiotic variables Y3 (2020/21)			
mod1_ab	retreat_response~ screen_type+eel_speed*turbidity	231	(null – mod1)
mod2_ab	retreat_response~ eel_speed*turbidity	231	(mod1-mod2)
Null_ab	retreat_response~ 1	258	-

Table S3. Alternative model selection (Generalised Linear Model; log-linked Gamma distribution) to determine non-tactile response distance (m) of European eels which had a non-tactile retreat from the pumping station. For each model the associated Akaiki Information Criteria (AIC) were calculated using glmmTMB in R 4.3.1. Light grey shading denotes model selected.

Model	Model formula	AIC	Model compared
Screen type + biotic variables Y2 – Y3 (2019/20 – 2020/21)			
mod1_b	nt_distance ~ screen_type*eel_speed+screen_type*eel_size	4.23	(null - mod1)
mod2_b	nt_distance ~ screen_type*eel_speed	1.96	(mod1- mod2)
mod3_b	nt_distance ~ screen_type+eel_speed	0.84	(mod2- mod3)
mod4_b	nt_distance ~ screen_type	8.73	(mod3- mod4)
mod5_b	nt_distance ~ eel_speed	5.84	(mod3- mod5)
mod6_b	nt_distance ~ eel_size	14.46	(mod2- mod6)
Null_b	nt_distance ~ 1	12.87	-
Screen type + abiotic variables Y3 (2020/21)			
mod1_ab	nt_distance screen_type+eel_speed*turbidity	-2.99	(null – mod1)
mod2_ab	nt_distance ~ eel_speed*turbidity	-2.07	(mod1-mod2)
Null_ab	nt_distance ~ 1	-2.09	-

Table S4. Results of Generalised Linear Mixed Models (GLMMs) examining the effects of pump duration, lunar phase and temperature on predicted count of night-time European eel approaches at the pumping station. GLMMs were fitted with log-linked zero-inflated negative binomial distributions and the random effect of month using package glmmTMB in R 4.3.1.

**GLMMs for night-time eel approach count**	**Coef est.**	**Std error**	**Z**	**Pr(>|z|)**
Y2 (2019/20)				
(Intercept)	-4.60479	1.04672	-4.399	<0.001
p1_both_duration	0.15479	0.03982	3.887	<0.001
moon_phase First Q	-0.09177	0.71333	-0.129	0.897
moon_phase Full moon	1.15366	0.50706	2.275	0.022
moon_phase Third Q	2.27706	0.44862	5.076	<0.001
avg_temp	0.34527	0.0934	3.697	<0.001
Y3 (2020/21)				
(Intercept)	-3.24998	0.76003	-4.276	<0.001
p1_both_duration	0.17549	0.02994	5.861	<0.001
moon_phase First Q	-0.07172	0.29293	-0.245	0.806
moon_phase Full moon	-0.53868	0.30942	-1.741	0.081
moon_phase Third Q	-0.93948	0.40249	-2.334	0.019
avg_temp	0.49891	0.07485	6.666	<0.001

Table S5. Results of Generalised Linear Mixed Models (GLMMs) examining the effects of weedscreen aperture, eel approach speed and turbidity on passage probability of European eels which approached the pumping station. GLMMs were fitted with logit-linked binomial distributions and the random effect of year (mod3_b) using package glmmTMB in R 4.3.1.

**GLMMs for passage probability**	**Coef Est.**	**Std error**	**Z**	**Pr(>|z|)**
Mod3_b (biotic) Y2 – Y4 (2019/20 – 2021/22)				
(Intercept)	-1.2491	0.2649	-4.716	< 0.001
screen_typeWide spacing	0.7330	0.1998	3.668	< 0.001
screen_typeFull screen wide	0.6433	0.2090	3.078	0.002
eel_speed	1.9134	0.7115	2.689	0.007
Mod1_ab (abiotic) Y3 (2020/21)				
(Intercept)	-0.2059	0.6866	-0.300	0.764
screen_typeWide spacing	0.8054	0.2400	3.355	< 0.001
eel_speed	-1.8895	1.9993	-0.945	0.344
turbidity	-0.0254	0.0179	-1.417	0.156
eel_speed:turbidity	0.0827	0.0491	1.684	0.092

Table S6. Results of Generalised Linear Mixed Models (GLMMs) examining the effects of weedscreen aperture, eel approach speed and turbidity on tactile behaviour probability of European eels which retreated from the pumping station. GLMMs were fitted with logit-linked binomial distributions and the random effect of year (mod2_b) using package glmmTMB in R 4.3.1.

**GLMMs for tactile behaviour probability**	**Coef Est.**	**Std error**	**Z**	**Pr(>|z|)**
Mod3_b (biotic) Y2 – Y4 (2019/20 – 2021/22)				
(Intercept)	2.1925	0.3739	5.864	<0.001
screen_typeWide spacing	0.1148	0.3345	0.343	0.731
screen_typeFull screen wide	2.0557	0.5734	3.585	<0.001
eel_speed	-2.5969	0.9969	-2.605	0.009
Mod1_ab (abiotic) Y3 (2020/21)				
(Intercept)	-1.5570	0.9623	-1.618	0.105
screen_typeWide spacing	0.4454	0.3956	1.126	0.260
eel_speed	3.3043	2.4976	1.323	0.185
turbidity	0.1751	0.0439	3.984	< 0.001
eel_speed:turbidity	-0.3006	0.0969	-3.099	0.001

Table S7. Results of Generalised Linear Models (GLMs) examining the effects of weedscreen aperture, eel approach speed and turbidity on non-tactile response distance (m) of European eels which had a non-tactile retreat from the pumping station. GLMs were fitted with log-linked Gamma distributions using package glmmTMB in R 4.3.1.

**GLM for non-tactile response distance**	**Coef Est.**	**Std error**	**Z**	**Pr(>|z|)**
Mod3_b (biotic) Y2 – Y3 (2019/20 – 2020/21)				
(Intercept)	-1.0648	0.1611	-6.610	< 0.001
screen_typeWide spacing	-0.3904	0.1322	-2.953	0.004
eel_speed	1.5287	0.4450	3.435	0.001
Mod1_ab (abiotic) Y3 (2020/21)				
(Intercept)	-0.8599	0.408148	-2.107	0.0409
screen_typeWide spacing	-0.2642	0.144915	-1.823	0.0751
eel_speed	1.4571	1.132843	1.286	0.2051
turbidity	-0.0081	0.022231	-0.366	0.7164
eel_speed:turbidity	-0.0138	0.057787	-0.24	0.8111

Table S8. Observed percentage passage (n passed / n retreated) for European eel that approached the 100-mm, 212-mm (1-m section) and 190-mm aperture weedscreen in each year and in total. – denotes were there are no data.

Year	100-mm	212-mm (1-m section)	190-mm
2018/19	40.6% (104/152)	-	-
2019/20	39.1% (45/70)	54.8% (23/19)	-
2020/21	33.1% (99/200)	53.6% (67/58)	-
2021/22	-	-	57.8% (107/78)
**Total**	**37.0% (248/422)**	**53.9% (90/77)**	**57.8% (107/78)**

Table S9. Observed percentage tactile response (n tactile / n non-tactile) for European eel that retreated from the 100-mm, 212-mm (1-m section) and 190-mm aperture weedscreens in each year and in total. – denotes where there ae no data.

Year	100-mm	212-mm (1-m section)	190-mm
2018/19	53.9% (82/70)	-	-
2019/20	75.7% (53/17)	84.2% (16/3)	-
2020/21	80.5% (161/39)	79.3% (46/12)	-
2021/22	-	-	94.9% (74/4)
**Total**	**70.1% (296/126)**	**80.5% (62/15)**	**94.9% (74/4)**

Table S10. Metrics used for multi-beam sonar (ARIS) analysis of European eel approach, passage, and retreat behaviour.

Metric	Description
**Date and time**	Date and time when eel entered the ARIS beam
**Size of eel (m)**	Length of eel manually measured in ARIS scope
**Passage**	Eel passed weedscreen in a downstream direction
**Retreat**	Eel did not pass the weedscreen and retreated in an upstream direction
**Tactile response**	Reaction (behavioural change) when eel contacted the weedscreen
**Non-tactile response**	Reaction (behavioural change) prior to weedscreen
**Retreat from the ASP**	Eel passed through the weedscreen in an upstream direction having already passed in a downstream direction
**Non-tactile response distance (m)**	Distance between weedscreen and position of response
**Speed of approach (m/s)**	Speed of approach prior to response

Table S11.Time series events used to analyse BDS data during pumping station passage.

Key event	Definition
**Injection**	Point when passage begins; pressure rise as pump chamber is entered
**Nadir**	Lowest pressure during passage
**Tailwater**	Point when pump passage is completed
**Pre-nadir time**	Time between injection and nadir
**Max post-nadir pressure**	Highest pressure after nadir and pre-tailwater
**Post-nadir time**	Time between nadir and tailwater
**Passage duration**	Total passage time; difference between injection and tailwater

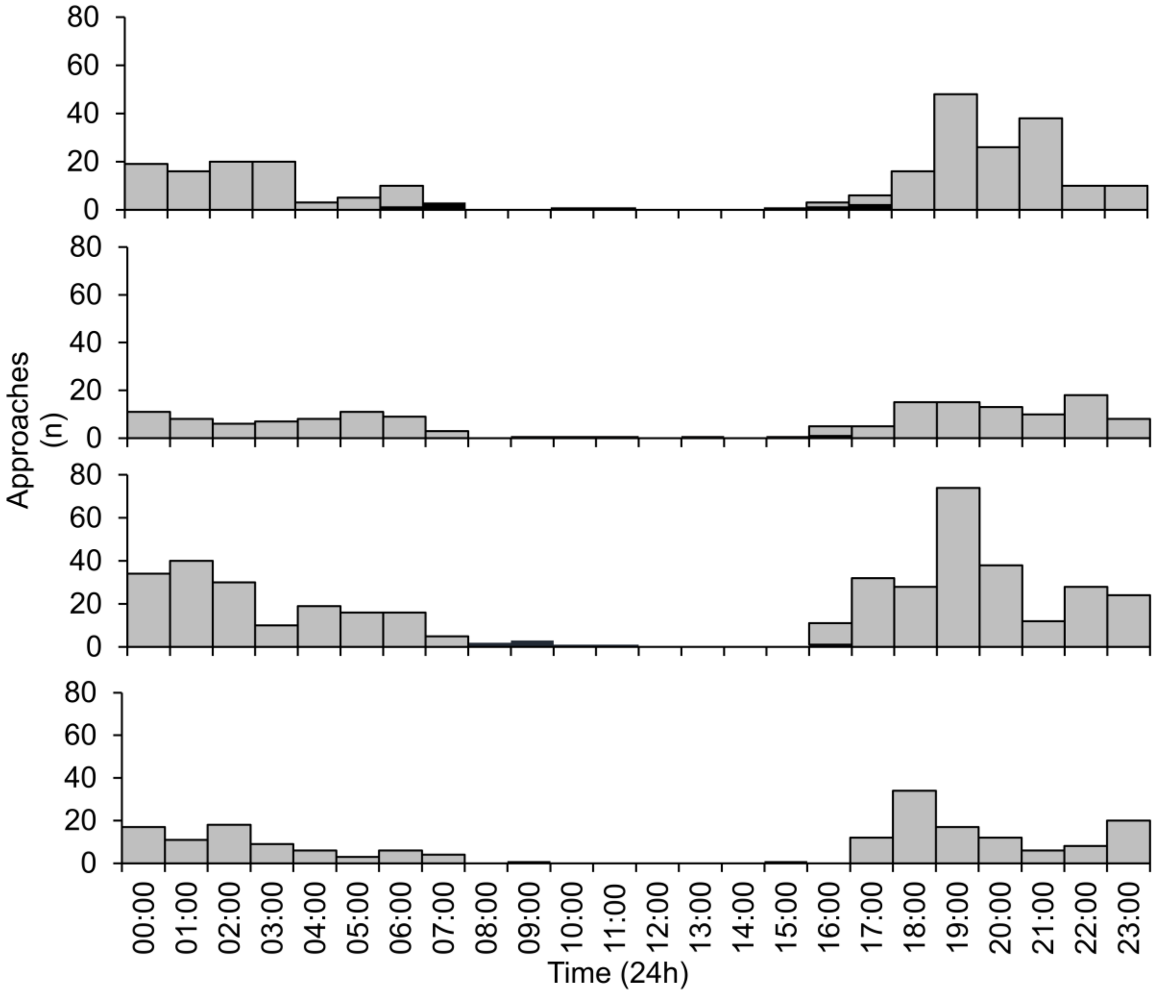

Figure S1. Number of eels that approached Bells pumping station each hour in 2018/19 (n = 256), 2019/20 (n = 157), 2020/2021 (n = 424) and 2021/22 (n = 185) (top to bottom), including whether during the day (black) or at night (grey).

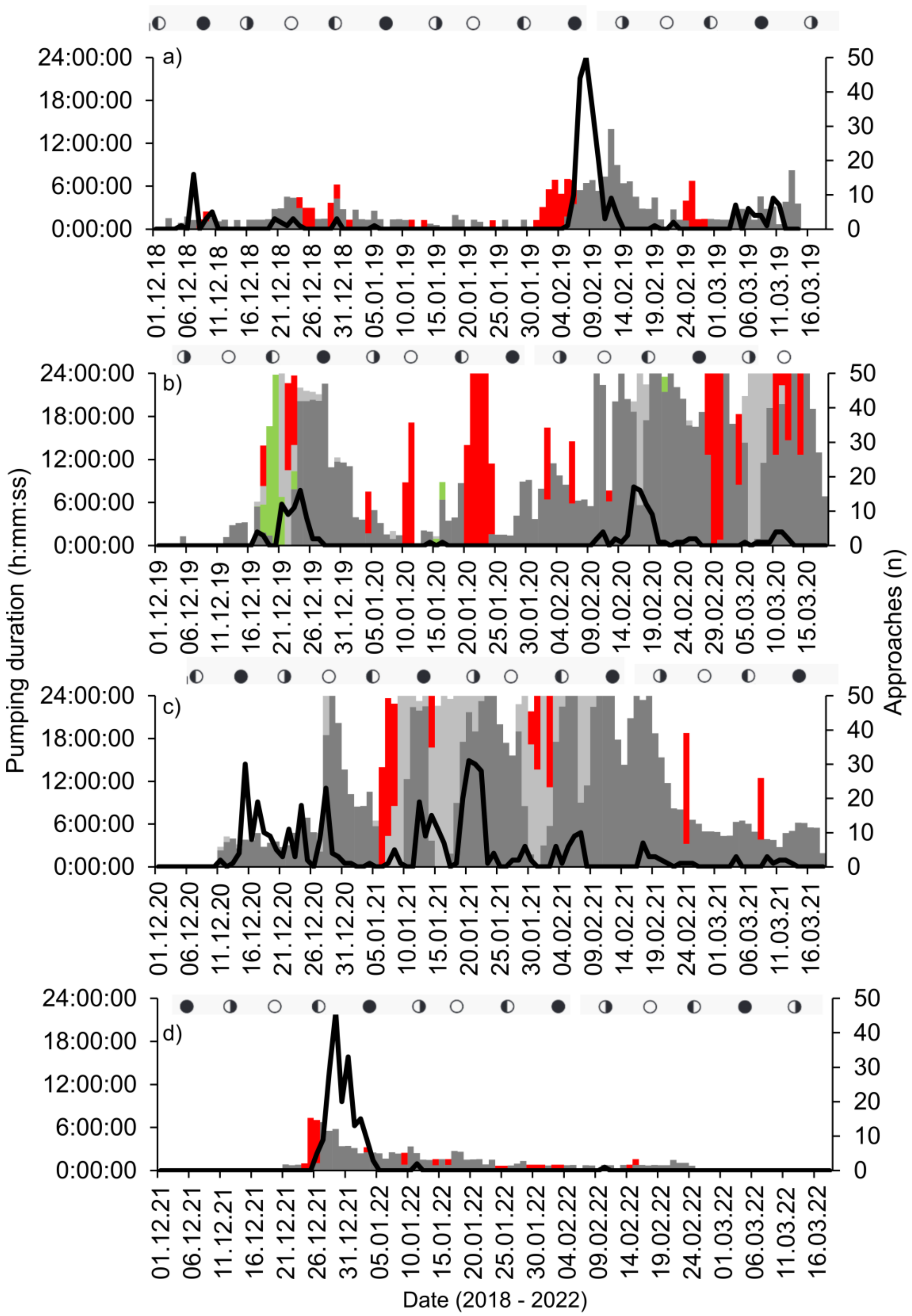

Figure S2. Number of eel that approached Bells pumping station each day (black line) in 2018/19 (n = 256), 2019/20 (n = 157), 2020/2021 (n = 424) and 2021/22 (n = 185) (a - d), including the number of hours Pump 1 (dark grey), Pump 2 (green) or both pumps (light grey) were running and when the Aris was not operational (red).
